# The neuroprotective effect of post ischemic brief mild hypothermic treatment correlates with apoptosis, but not with gliosis in endothelin-1 treated rats

**DOI:** 10.1186/1471-2202-13-105

**Published:** 2012-08-26

**Authors:** Tine Zgavc, An-Gaëlle Ceulemans, Said Hachimi-Idrissi, Ron Kooijman, Sophie Sarre, Yvette Michotte

**Affiliations:** 1 Department of Pharmaceutical Chemistry and Drug Analysis, Center for Neuroscience, Vrije Universiteit Brussel, Laarbeeklaan 103, Brussels, Belgium; 2 Critical Care Department and Cerebral Resuscitation Research Group, Center for Neuroscience, Vrije Universiteit Brussel, Laarbeeklaan 103, Brussels, Belgium; 3 Department of Pharmacology, Center for Neuroscience, Vrije Universiteit Brussel, Laarbeeklaan 103, Brussels, Belgium

**Keywords:** Hypothermia, Cerebral ischemia, Endothelin-1, Caspase-3, Gliosis, Phagocytosis

## Abstract

**Background:**

Stroke remains one of the most common diseases with a serious impact on quality of life but few effective treatments exist. Mild hypothermia (33°C) is a promising neuroprotective therapy in stroke management. This study investigated whether a delayed short mild hypothermic treatment is still beneficial as neuroprotective strategy in the endothelin-1 (Et-1) rat model for a transient focal cerebral ischemia. Two hours of mild hypothermia (33°C) was induced 20, 60 or 120 minutes after Et-1 infusion. During the experiment the cerebral blood flow (CBF) was measured via Laser Doppler Flowmetry in the striatum, which represents the core of the infarct. Functional outcome and infarct volume were assessed 24 hours after the insult. In this sub-acute phase following stroke induction, the effects of the hypothermic treatment on apoptosis, phagocytosis and astrogliosis were assessed as well. Apoptosis was determined using caspase-3 immunohistochemistry, phagocytic cells were visualized by CD-68 expression and astrogliosis was studied by glial fibrillary acidic protein (GFAP) staining.

**Results:**

Cooling could be postponed up to 1 hour after the onset of the insult without losing its positive effects on neurological deficit and infarct volume. These results correlated with the caspase-3 staining. In contrast, the increased CD-68 expression post-stroke was reduced in the core of the insult with all treatment protocols. Hypothermia also reduced the increased levels of GFAP staining, even when it was delayed up to 2 hours after the insult. The study confirmed that the induction of the hypothermia treatment in the Et-1 model does not affect the CBF.

**Conclusions:**

These data indicate that in the Et-1 rat model, a short mild hypothermic treatment delayed for 1 hour is still neuroprotective and correlates with apoptosis. At the same time, hypothermia also establishes a lasting inhibitory effect on the activation of astrogliosis.

## Background

Stroke is an important cause of death worldwide and recombinant tissue plasminogen activator remains the only approved treatment [1]. However, the limited time window for its administration restricts its usefulness. Given the numerous pathways via which ischemia causes cell death, the capacity to inhibit multiple mechanisms simultaneously may provide additive clinical benefits for stroke patients [2,3]. Therapeutic hypothermia has given positive effects in cardiac arrest and newborns with hypoxic-ischemic encephalopathy [2,4,5]. A number of small clinical trials have been performed in stroke patients subjected to hypothermia, such as the “Cooling for Acute Ischemic Brain Damage” study and the “Intravascular Cooling in the Treatment of Stroke–Longer tPA window” study [6,7]. Both studies confirmed that cooling patients is feasible. However, no reduced mortality could be shown. Several studies in animals have shown that although brief durations of pre-insult hypothermia may be sufficient to protect against cerebral ischemia, longer durations are necessary when started in the post-ischemic period [8,9]. However, the risk of complications increases with treatment duration, such as pneumonia hypovolemia, arrhythmias, hyperglycemia, bradycardia, thrombocytopenia, hypertension, hypotension, increased intracranial pressure, electrolyte abnormalities like hypokalemia and metabolic acidosis [7,10-13]. Evidently, hypothermic treatments should be as short as possible. Indeed, with respect to the well-known *time is brain* concept, it is crucial to induce a beneficial effect as early as possible and to start a treatment with as little side-effects as possible. Therefore, it is essential that the ideal therapeutic time window of a short hypothermic treatment should be determined in order to avoid long cooling times and this without further impairment of the neurological outcome. Many have proven that 2 hours of mild hypothermia is neuroprotective and can reduce infarct volume after MCAO [3,9,14-26]. Experimental research remains vital to establish the ideal length of the therapeutic hypothermic treatment and this without increasing hypothermia related complications [13,27-29]. As there is no ideal experimental stroke model, it is essential that such parameters are investigated in various animal models mimicking different types of stroke. In contrast to other Middle Cerebral Artery Occlusion (MCAO) models, the endothelin-1 (Et-1) model allows the study of the effects of cooling during a slow reperfusion phase [20,30-33]. Although longer cooling times (minimum 12 hours) in rat models seem more protective [34,35] than short ones (a few hours), investigation into efficient shorter cooling strategies remains relevant as long hypothermic treatments may increase the risk of complications and/or have an influence on different organ systems [13,36]. We previously showed that a 2 hours mild hypothermic treatment, started 20 minutes after the onset of the insult can reduce infarct volume up to 1 week after an Et-1 induced stroke. This beneficial effect was related to effects on apoptosis, oxidative stress and the inflammatory response [20,21,37].

Here, we investigated how long a short mild hypothermic treatment can be postponed without reducing its neuroprotective effect after a transient cerebral ischemia induced by Et-1. The study investigated first whether a hypothermia treatment exerts an effect on the cerebral blood flow (CBF). Secondly, since apoptosis and neuroinflammation are important pathways in cell death in the subacute phase after ischemia, activated caspase-3, phagocytosis (CD-68 expression) and astrogliosis (glial fibrillary acidic protein (GFAP) staining) were investigated. The effects were assessed after 24 hours since the most pronounced effects of this hypothermic treatment on apoptosis, phagocytosis and astrogliosis occur at that time point.

## Methods

The experiments were performed according to the National Guidelines on Animal Experimentation and approved by the Ethical Committee for Animal Experimentation of the Faculty of Medicine and Pharmacy of the Vrije Universiteit Brussel.

### Experimental and surgical protocols

Experiments were carried out in male Wistar rats weighing 270-300 g (Charles River Laboratories, IFFA-CREDO, Germany). A day before the induction of the insult, 2 intracerebral guide cannulas were stereotactically implanted under anaesthesia (ketamine/diazepam 75:4 mg/kg i.p.) [21,37]. An Et-1 administration probe was positioned close to the middle cerebral artery (MCA) (relative to bregma: AP +0.9 mm; L +5.0 mm; V +2.8 mm) and a thermocouple probe was positioned in the contralateral prefrontal cortex (AP +3.2 mm; L −3.0 mm; V +2.3 mm) [38]. As post-operative analgesia, the rats received 4 mg/kg ketoprofen (i.p.). After surgery, the rats were allowed to recover overnight. The next day the animals were anaesthetized with 4% sevoflurane (Sevorane®, Abbott, Kent, England) and oxygen insufflated into a transparent chamber. During the experiments, anaesthesia was maintained by 2% sevoflurane with oxygen at 0.8 ml/min via a facemask. The guides were replaced by a microdialysis probe without a membrane (CMA, 3 mm probe with removed membrane, Stockholm, Sweden) and a thermocouple probe (HYP-O-SLE, Omega Corporation, Stamford, USA) to infuse Et-1 and measure the brain temperature respectively. Heart rate, oxygen saturation and temperature were continuously monitored during the experimental procedure. Transient focal cerebral ischemia was induced by infusion of Et-1 (Sigma, St-Louis, MO, USA) dissolved in Ringer’s solution (500pmol/6 μl) through the probe near the MCA at a flow rate of 1 μl/min. In sham experiments, only Ringer’s solution was injected. By infusing Et-1 adjacent to the MCA, a reproducible insult can be obtained in which the core is represented by the striatum and the penumbra by the surrounding cortex [21,37]. In normothermic rats, the brain temperature was maintained at 37.0 ± 0.5°C throughout the experiment using a heating pad and an infrared lamp. In the hypothermic group, the temperature was reduced to 33.0 ± 0.5°C during 2 hours starting with a delay of 20, 60 or 120 minutes after the ischemic insult. Cooling the animal to the target temperature was achieved by spraying alcohol onto the animal and cooling it with a fan. With this method the brain temperature can be decreased from 37.0 to 33.0°C within 10 minutes. A heating pad and an infrared lamp were used to re-warm the animal from 33.0 to 37.0°C in 30 minutes. Finally, all rats were kept at 37°C for another 30 minutes before the anaesthesia was stopped. Laser-doppler flowmetry experiments were preformed in the striatum of some animals to investigated the influence of cooling on the MCA occlusion caused by Et-1. Twenty-four hours after the induction of the ischemic insult, the effects on neurological outcome (neurological deficit score), infarct volume, apoptosis (active caspase-3 staining) and activation of microglia and astrocytes (CD-68 and GFAP expression, respectively) were evaluated.

### Experimental groups

**Figure 1:**
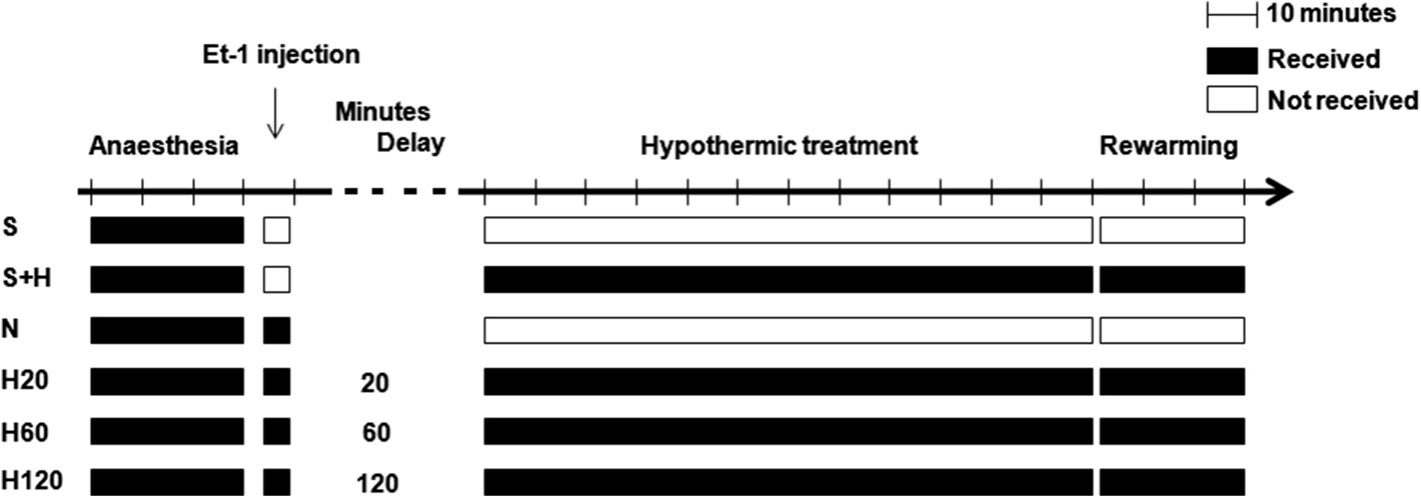
Schematic overview of the different experimental groups included in this study.

In total, 44 rats were randomized into 6 experimental groups (Figure 1). Sham rats (S group) were injected with the vehicle of Et-1 and the brain temperature was kept constant at 37.0 ± 0.5°C (n = 7) while in sham rats that received the hypothermic treatment (S + H group, n = 4), brain temperature was reduced to 33.0 ± 0.5°C. Normothermic rats (N group) were injected with Et-1. These animals were not cooled and their brain temperature was maintained at 37.0 ± 0.5°C (n = 12). Hypothermic rats were infused with Et-1 and to determine how long the hypothermic treatment protocol could be delayed, cooling was started 20 (H20 group, n = 7), 60 (H60 group, n = 7) or 120 minutes (H120 group, n = 7) after the infusion of Et-1. As rats subjected to the 2 hours delayed hypothermic protocol had to be sedated 100 minutes longer than the H20 hypothermic protocol, we first investigated if such prolonged anaesthesia did not affect the parameters studied. Therefore, an extra group of normothermic rats was included in the study that received prolonged sedation (100 minutes extra). The results showed no differences in the determined parameters when rats were subjected to the prolonged protocol. Therefore, the results of all normothermic animals were pooled and are always referred to as the N group.

### Cerebral blood flow measurement

In 12 animals the CBF was measured during the entire experimental procedure using laser Doppler flowmetry (S, N and H group; n = 4 in each group). For these experiments, an extra guide with canula was inserted into the striatum during the surgical procedure (AP +1.2, L +2.4, V +5.8, coordinates relative to bregma) [38]. The next day when performing the experiment as described above, this guide was replaced by a laser Doppler probe (LaserFlo Blood perfusion monitor 403A, fiber optic probe P336387). This allowed continuous measurement of the CBF during the entire experimental procedure. Basal levels of the CBF were equalled to 100% [39].

### Behavioural testing

Sensor- and motoric abnormalities were evaluated before and 24 hours after the administration of Et-1 with the use of a neurological deficit score (NDS) as previously described [37]. Six parameters were scored between 0 to 3 or 1 to 3. Spontaneous activity, symmetry in the movement of the 4 limbs, forepaw outstretching, equality of strength in the forepaws, body proprioception and response to vibrissae touch was observed. The NDS was calculated as the sum of these scores, 18 being the best and 3 the worst possible score [37,40]. To ascertain unbiased results, behavioural testing was performed by observers, blinded to the treatment protocol.

### Infarct volume determination

Exactly 24 hours after the infusion of Et-1, after the behavioural analysis, rats were deeply anaesthetized with 6% pentobarbital. The brains were fixated by a transcardial perfusion with 5 minutes of saline and 5 minutes of a freshly prepared 4% phosphate buffered paraformaldehyde solution and then stored in the same buffer. Coronal sections of 50 μm were cut and stored in phosphate buffered saline (PBS, 0.01 M) containing sodium azide (0.1%) as a preservative. The infarct area was visualized every 200 μm by a Nissl staining. At a magnification of 1.25, microscope pictures of the stained slices were taken and transferred to the computer program Image J (NIH, version 1.37) to calculate the infarct volume stereologically. After scaling, the marked infarcted area and hemisphere (in mm^2^) could be calculated and was multiplied with the interspace. The influence of oedema on the infarct volume was corrected by applying the following formula: (area of normal hemisphere/area of infarcted hemisphere) x area of infarct [37,41]. To ascertain unbiased results, determination of the infarct volume was performed by observers, blinded to the treatment protocol.

### Immunohistochemistry (IHC) for active caspase-3, CD-68 and GFAP

The degree of *apoptosis* was determined by IHC for active caspase-3^+^-cells as previously described but with a slightly modified protocol [20]. The 50 μm sections were pre-incubated with 0.01% Triton X-100, 3% hydrogen peroxide and blocking agent (Vectastain ABC kit, VectorLaboratories, Burlingame, CA, USA). Next, the sections were incubated overnight at 4°C with a 1:250 dilution of the primary monoclonal rabbit anti-caspase-3 antibody (Cell Signaling, Westburg, The Netherlands). The next day, the slices were incubated for 1 hour at room temperature with biotinylated secondary antibody (Vectastain ABC kit, VectorLaboratories, Burlingame, CA, USA) and 30 minutes with ABC reagent with blocking solution (Vectastain ABC kit, VectorLaboratories, Burlingame, CA, USA). Antibody binding was visualized using the diaminobenzidine substrate chromogen kit (DakoCytomation, Glostrup, Denmark). Between all incubations, a washing step was included. After drying, the sections were counterstained with haematoxylin/lithium carbonate.

To determine the degree of *neuroinflammation,* the activation status of microglia (CD-68 expression) and astrocytes (GFAP expression) was determined as previously described [37]. After pre-incubation with 0.01% Triton X-100, 3% hydrogen peroxide and pre-immunized goat serum (1:5 dilution, Sigma, St-Louis, MO, USA), brain slices were incubated overnight at 4°C, with polyclonal mouse anti-CD-68 (1:1000 in PIG/PBS 1:5 dilution, AdB Serotec, Düsseldorf, Germany) or polyclonal rabbit anti-GFAP (1:10000 in PBS, DakoCytomation, Glostrup, Denmark). Next, the slices were incubated for 4 hours at room temperature with a 1:100 dilution of either sheep anti-mouse or monkey anti-rabbit secondary antibody (Amersham, GE Healthcare, Buckinghamshire, UK). Antibody binding was visualized using the diaminobenzidine substrate chromogen kit (DakoCytomation, Glostrup, Denmark). Between all incubations, a washing step was performed. After drying, the sections were washed with water and dehydrated by passing to graded alcohol and xylene.

For each rat, IHC protocols were performed on 3 independent slices taken from 0.2 mm anterior to 0.26 mm posterior to bregma to avoid interference of the implantation of the Et-1 probe [38]. Alternate sections were used for the determination of the infarct volume, apoptosis or activation of glial cells. Positive caspase-3 stained cells were counted using standard light microscopy. As previous research showed that hypothermia reduces the degree of apoptosis only in the cortex after infusion of Et-1, but not in the striatum, counting of active caspase-3^+^-cells was limited to the cortex [20,21]. The total number of caspase-3 positive cells was counted in the entire cortex of the ischemic hemisphere. CD-68 positive cells were counted in striatum and cortex [22,37]. For GFAP expression, the high density of cells did not allow counting. Therefore, the relative staining intensity was calculated in each rat: mean gray value ipsilateral striatum (or cortex) – mean gray value contralateral striatum (or cortex), using Image J (NIH, version 1.37). Besides, calculating the difference in intensity between the ipsi- and the contralateral side is advantageous to correct for variations in circumstances when the IHC protocols were performed. To ascertain unbiased results, counting was performed by observers, blinded to the treatment protocol.

### Correlation between the number of apoptotic cells, the infarct volume and the NDS

The correlation between the number of apoptotic cells, the infarct volume and the NDS was investigated. Each point represents the mean of the S, N, H20, H60 and H120 group. The calculated r^2^ values represent the coefficient of determination (square of the Pearson correlation coefficient) and are a measure for the extent to which a variance in the two variables is shared.

### Statistical analysis

All data are expressed as mean ± SEM and a p-value < 0.05 was considered significant. Data analysis was performed using the statistical program Graphpad InStat (version 3.06 Windows XP, GraphPad Software, San Diego, California, USA). To compare the results between the different groups, a one-way ANOVA with Bonferroni post-hoc test was used. For the changes of the CBF as a function of time in each group, a repeated measures ANOVA was used with Dunnett post-hoc test compared to the CBF before the injection of Et-1.

## Results

### Mild hypothermia has no effect on the CBF

**Figure 2:**
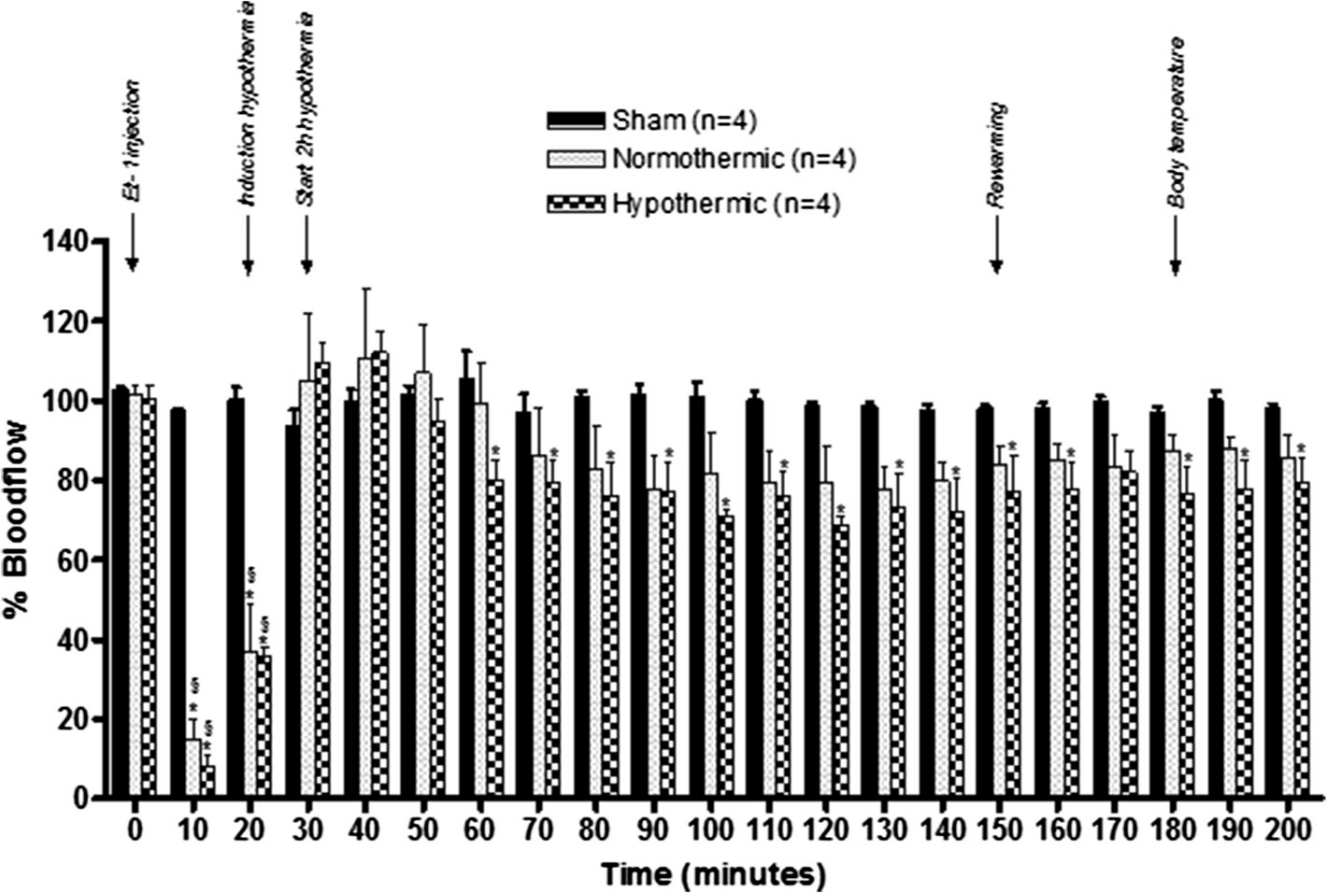
**Effect of the hypothermic treatment on the CBF after injection of Et-1.** The graph shows the percentage increase or decrease in CBF in sham, normothermic or hypothermic animals (n = 4 in each group), compared to basal values (equal to 100%) which were assessed before injection of Et-1 or vehicle. The hypothermia treatment in the Et-1 model does not affect the CBF. *Significantly different (p < 0.05) from the time point 0 min. ^§^Significantly different (p < 0.05) compared to the sham group.

Sham animals showed no change in CBF during or after administration of the vehicle. In normothermic conditions, administration of 500 pmol Et-1 (at t = 0 minutes) resulted in an immediate significant reduction in CBF for 20 minutes to approximately 20% of the baseline values. Forty minutes after the insult, a slight hyperperfusion phase could be distinguished during which the CBF increased to about 110% of baseline values. Gradually, however, the CBF decreased back to approximately 75% of basal values. The hypothermic treatment, started 20 minutes after the Et-1 administration, had no effect on the changes in blood flow observed in normothermic conditions. So, induction of hypothermia in our model does not affect the CBF (Figure 2).

### Delaying hypothermia up to 1 hour after the administration of Et-1 is still neuroprotective and improves functional and neurological outcome

**Figure 3:**
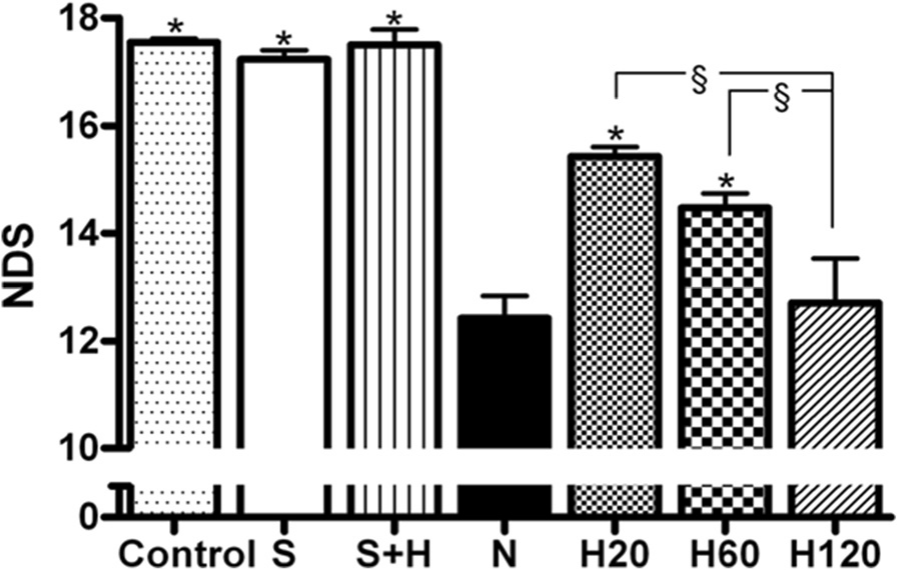
**Effect of hypothermic treatments on the Et-1 induced changes in neurological deficit score (NDS).** The S and S + H group showed no significant decrease in NDS (n = 7 and 4 respectively). Delaying hypothermia (n = 7 in each H group) up to 60 minutes after the induction of the insult, still improved neurological deficit. *Significantly different (p < 0.05) from N group (n = 12). ^§^Significantly different (p < 0.05) from each other.

The minor surgery required to implant the probe by which Et-1 was administered, had no effect on the NDS (data not shown). Comparison of the NDS before infusion (control) of vehicle in the sham rats and 24 hours afterwards showed no significant difference. The NDS of the S + H group was not different from the control group. Et-1 administration in normothermic conditions significantly reduced the NDS compared to the sham rats. The NDS in the H20, 60 or 120 group was also lower than in the S group. The hypothermic treatment initiated either 20 or 60 minutes after the ischemic insult significantly improved the neurological deficit compared to the normothermic group, whereas the treatment initiated after 120 minutes was ineffective (Figure 3).

**Figure 4:**
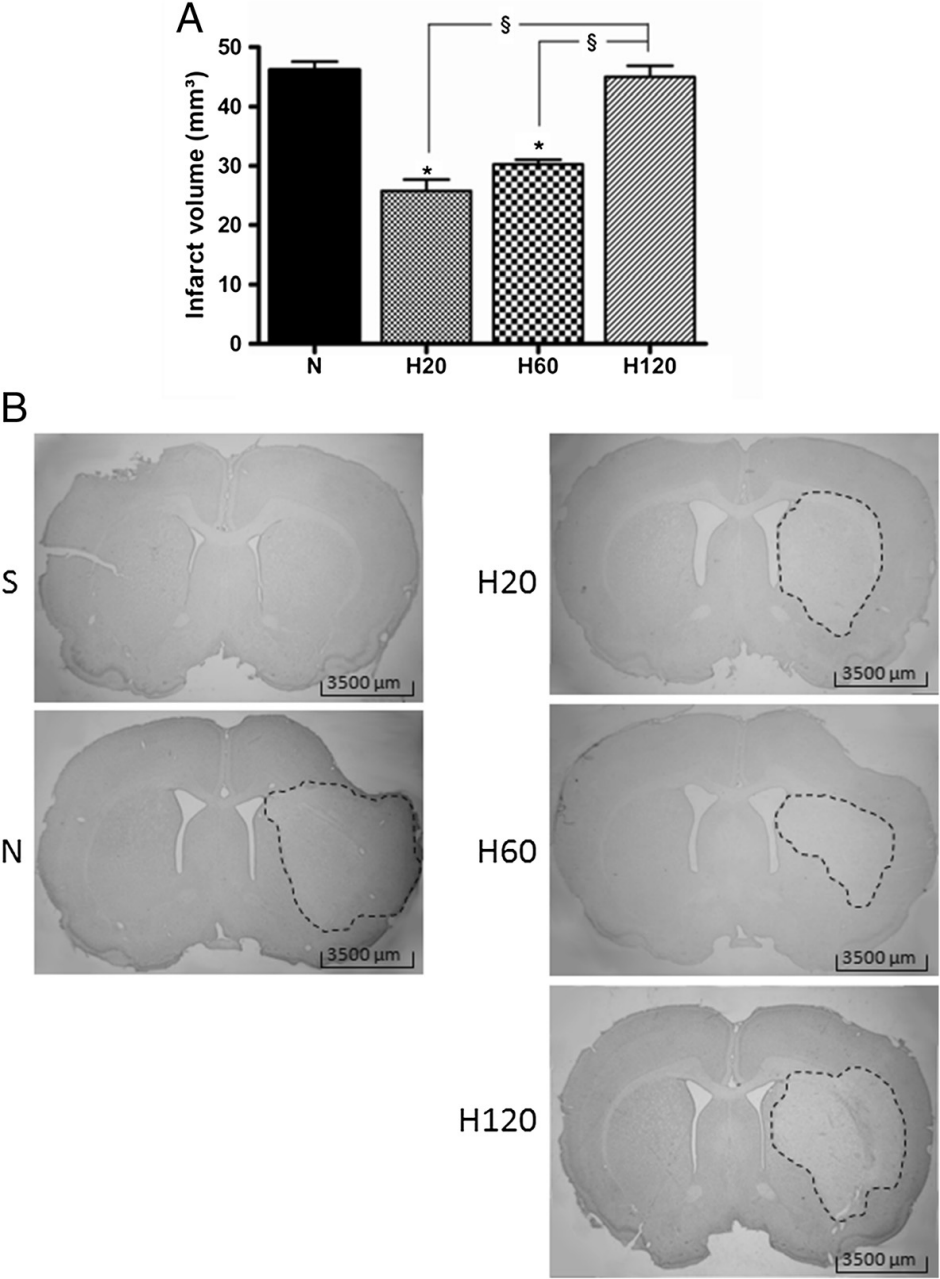
**Effect of hypothermic treatments on the Et-1 induced infarct volume.** There was no infarct volume in the S and the S + H group (n = 7 and 4 respectively, data not shown). (**A**) Delaying hypothermia up to 60 minutes after the administration of Et-1 still induced enough neuroprotection to reduce the infarct volume significantly. The amount of animals in each hypothermic group was 7. *Significantly different compared to the infarct volume of the N group (n = 12). ^§^Significantly different from each other (p < 0.05). (**B**) shows the infarct volume for the different treatment protocols.

Sham operated animals (S and the S + H group) showed no infarct, while in the normothermic rats a large area of damaged tissue stretching from the striatum (the core of the insult) into the surrounding cortex (the penumbra) was present. Hypothermia, initiated 20 minutes after the insult reduced the infarct volume by almost half. Salvation of tissue by hypothermia predominantly took place in the penumbral region, in accordance with previous findings from our laboratory [21,37]. Hypothermia, initiated 60 minutes after the ischemic insult, also significantly reduced the infarct volume. However, when the hypothermic treatment was delayed up to 2 hours after the insult, the infarct volume did not show any significant improvement compared to normothermic rats anymore (Figure 4). 

### Hypothermia reduces the amount of active caspase-3^+^-cells by half, even if only initiated 1 hour after the administration of Et-1

**Figure 5:**
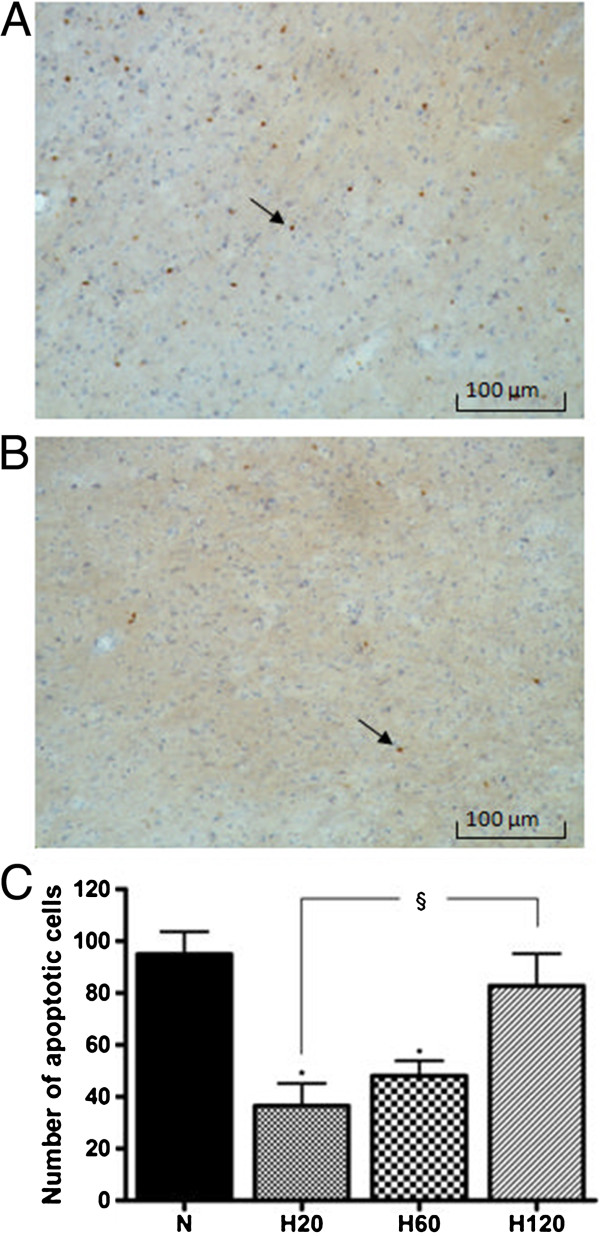
**Effect of hypothermic treatments on the Et-1 induced changes in active caspase-3**^**+**^**-cells in the cortex.** (**A**) shows the active caspase-3^+^-cells of a normothermic rat, while (**B**) in a hypothermic animal. Graph (**C**) shows the overall expression of active caspase-3^+^-cells of the N group (n = 12) and the H20, H60 and H120 group, (n = 7 in each group), in the ischemic hemisphere. There was no staining in the S and the S + H group (n = 7 and 4 respectively, data not shown), and in the contralateral hemisphere of the other groups. Delaying hypothermia up to 60 minutes after the administration of Et-1, reduced the Et-1 induced apoptosis significantly. There was no binding of the second antibody in the absence of the primary antibody (data not shown). *Significantly different compared to the N group. ^§^Significantly different (p < 0.05) from each other.

No positive staining was observed in sham rats (S and S + H group) and in the hemisphere contralateral to the site of Et-1 infusion. Administration of Et-1, on the other hand, resulted in a massive increase in apoptotic cell death (Figure 5). The hypothermic treatment initiated 20 or 60 minutes after the insult resulted in a 50 or 45% reduction in apoptotic cells respectively. However, starting hypothermia as late as 2 hours after the Et-1 administration did not reduce the amount of apoptotic cells significantly, compared to normothermic rats.

### The number of apoptotic cells correlates well with the infarct volume and the NDS

**Figure 6:**
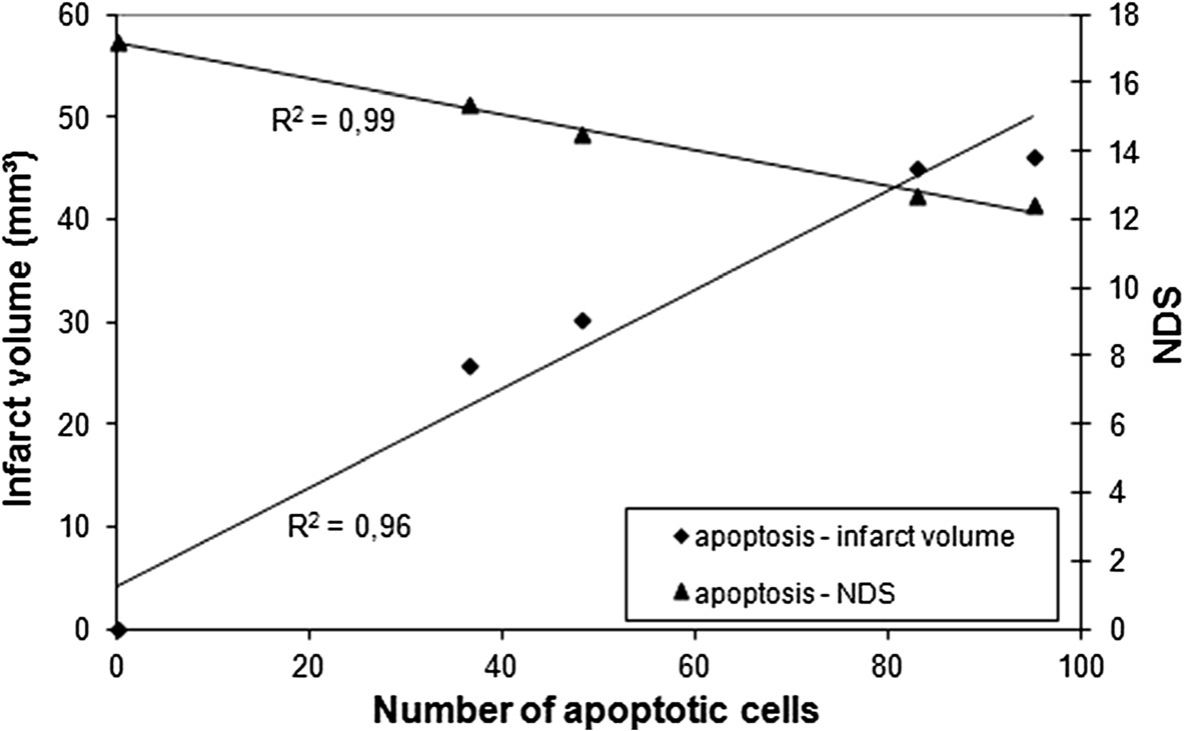
**Correlation between the number of apoptotic cells and the infarct volume, and the NDS.** Each point represents the mean of the S, N, H20, H60 and H120 group. The number of apoptotic cells correlates well with infarct volume (r^2^ = 0.96) and with the NDS (r^2^ = 0.99).

The number of apoptotic cells correlated well with infarct volume (r^2^ = 0.96) and even better with the NDS (r^2^ = 0.99) (Figure 6). The NDS also correlated well with the infarct volume (r^2^ = 0.96) (data not shown).

### Delaying hypothermia up to 2 hours after stroke onset, still reduces CD-68 expression but not GFAP expression

**Figure 7:**
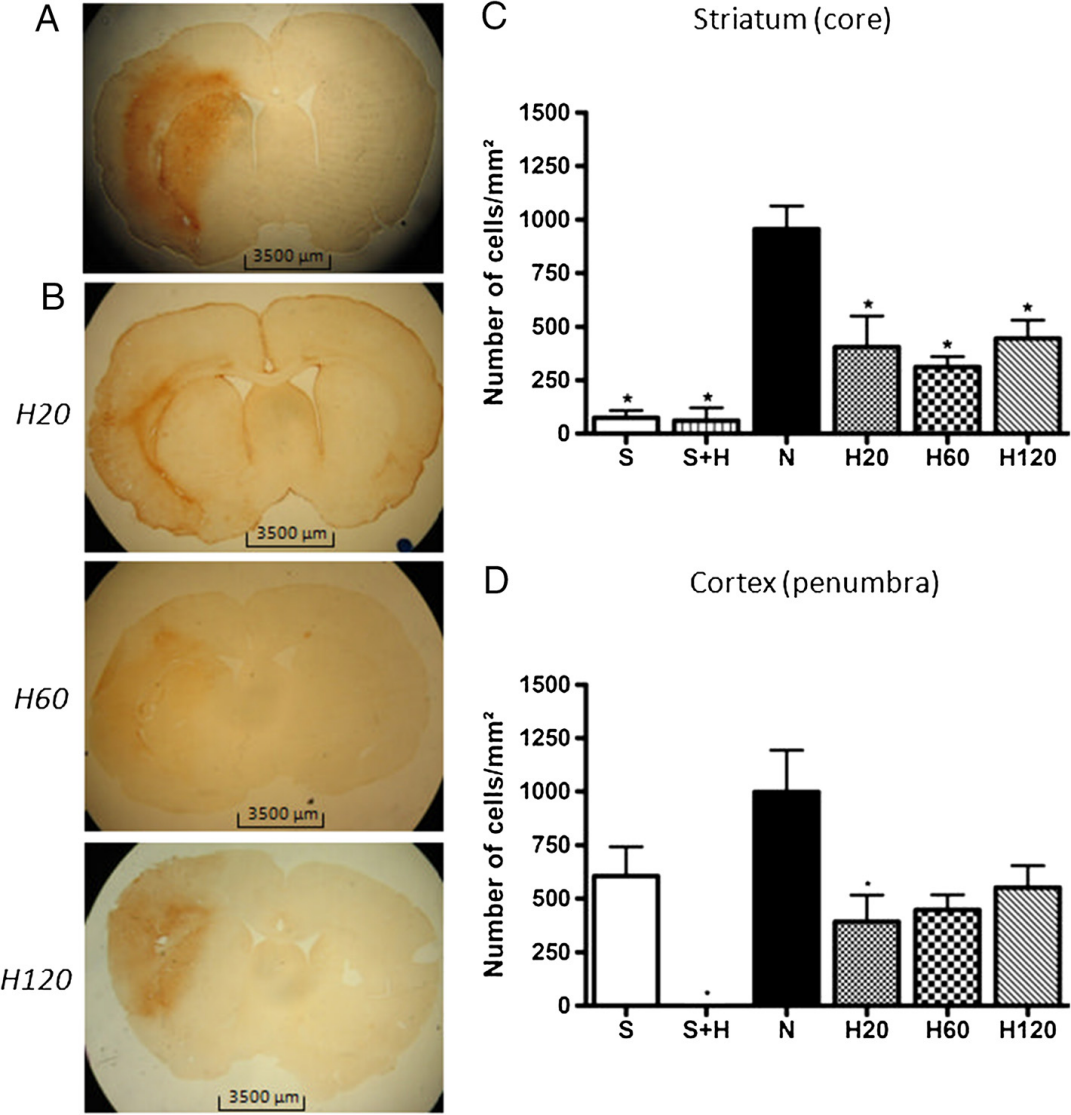
**Effect of hypothermic treatment on the Et-1 induced increase in CD-68 expression.** (**A**) shows the expression of CD-68 in a rat of the N group and (**B**) in a rat of the H20, H60 and H120 group, respectively. The graphs show the overall expression in the striatum (**C**) and the cortex (**D**) for the different treatment protocols. The number of rats in the S and S + H group is 7 and 4 respectively; in the N group 12 and in every H group 7. Data are expressed as the number of cells/mm^2^. There was no binding of the second antibody in the absence of the primary antibody (data not shown). *Significantly different compared to the N group. For a more detailed image we refer to a previously published article by Ceulemans et al. [37].

Cerebral ischemia induces neuroinflammation which is initiated by activation of microglia and their transformation into resident macrophages and subsequent infiltration of leukocytes, including neutrophils and macrophages. Blood-borne and resident macrophages/activated microglia can be visualized by anti-CD-68 staining. No positive staining was observed in the hemisphere contralateral to the site of Et-1 infusion. Sham operated rats showed a low number of CD-68^+^-cells in the striatum, in contrast to the cortex, where a significant amount of CD-68^+^-cells was observed. Cooling down sham rats reduced the expression of CD-68 in the cortex. Administration of Et-1 resulted in an increased CD-68 expression in striatum and cortex, in accordance with previous findings [37]. The hypothermic treatment, even if delayed up to 2 hours after the infusion of Et-1 significantly reduced the amount of phagocytic cells in the striatum. However, in the cortex, although a strong reduction in CD-68^+^-cells was visible after all treatment protocols, this effect was only statistically significant for the H20 group (Figure 7). 

**Figure 8:**
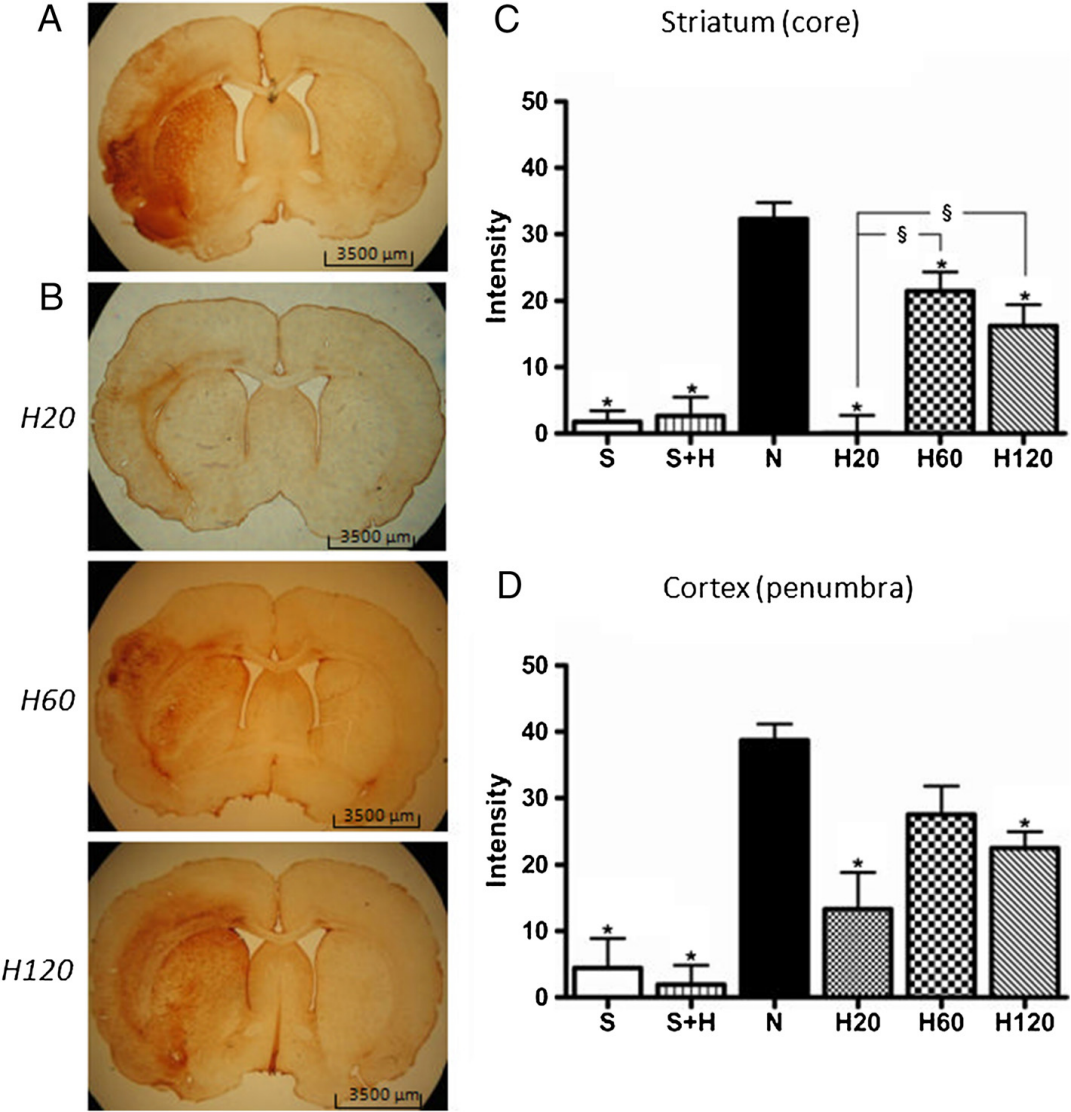
**Effect of hypothermic treatment on the Et-1 induced increase in GFAP expression.** Activation of astrocytes as determined by the expression of GFAP (**A**) in a N rat and (B) in a H20, H60 and H120 rat, 24 hours after the administration of Et-1. The graphs show the overall expression in striatum (**C**) and cortex (**D**) of the different treatment protocols. The number of rats in the S and S + H group is 7 and 4 respectively; in the N pool 12 and in every H group 7. *Significantly different compared to the N group. ^§^Significantly different (p < 0.05) from each other. For a more detailed image we refer to a previously published article by Ceulemans et al. [37].

Activated astrocytes were visualized by IHC for GFAP. Low expression of GFAP was present in the striatum and the cortex of the sham rats (S and S + H group). Consistent with our previous findings, a massive increase in GFAP expression occurred in the striatum and cortex of the normothermic animals. Hypothermic treatment after Et-1 significantly reduced the GFAP expression even when hypothermia was delayed for 2 hours after the insult. The strongest reduction in GFAP expression was observed in the striatum of the H20 group. The levels of the H60 group were higher than the H20 group, but stayed significantly lower than the untreated N group. The same was seen in the H120 group but the levels decreased again slightly compared to the H60 group, although not significantly different from each other. Reduction in GFAP expression was also observed in the cortex but was less pronounced than in the striatum (Figure 8).

## Discussion

It is generally accepted that reducing the body temperature to 33-34°C is neuroprotective against cerebral ischemic insults without causing many side effects [9,28,29]. Here, we show that, in the Et-1 model, a short mild hypothermic treatment can be delayed up to 1 hour after stroke onset without losing its beneficial effect. Although the reduction in neurological and functional deficit and apoptosis was no longer observed after delaying hypothermia for 2 hours, this study clearly shows a lasting inhibitory effect on activation of glia. Laser-doppler flowmetry experiments in the striatum showed no influence of cooling on the MCA occlusion caused by Et-1. Finally, we show a clear correlation between neurological and functional outcome with apoptosis but not with inflammation.

The STAIR criteria suggest to reproduce data in as many experimental stroke models as possible, especially in models mimicking hospital settings. In the Et-1 model, as reperfusion is only established slowly, it resembles clinical reality closely and thus data could be extrapolated to the clinic [42]. Besides, due to the short half-life of Et-1 (1.4 to 3.6 minutes) [43], infusion of this vasoconstrictor leads to an occlusion (>75%) of the MCA for 20–30 minutes, before a gradual reperfusion [39]. Afterwards the blood flow is restored. As such, a reproducible infarct with a clear distinction between the core (striatum) and the penumbra (cortex) of the insult is established and it becomes possible to distinguish certain effects solely to the core or penumbral area [21,37]. Our study showed that cooling had no influence on the MCAO caused by Et-1. This result indicates that the effects of hypothermia are not mediated by modulation of the CBF.

Since cells in the penumbra undergoing apoptosis are major targets for intervention [44], we assessed the number of cells containing active caspase-3. Previously, we demonstrated that apoptosis in the penumbra occurs in the subacute phase and peaks 24 hours after the insult [20]. Chaitanya et al. [45] showed that 3 hours MCAO induces apoptosis, reaching maximal levels at 24 and 72 hours after the insult [45]. Another study investigated the effect of 20 minutes and 2 hours MCAO on apoptosis. They observed that caspase-3 activity peaks at 24 and 72 hours after reperfusion in the 20 minutes MCAO group, whereas, it only peaks at 24 hours after 2 hours MCAO [46]. These results are consistent with our data. Previous research in our laboratory also investigated nuclear fragmentation in neuronal cells and showed 24 hours after the insult that mild hypothermia significantly affected apoptotic neuronal cell death in the penumbral region, consistent with the effects observed here on activated caspase-3 expression [21]. Phanithi et al. [47] showed that intra-ischemic mild hypothermia inhibits the caspase-3 expression in the penumbra, at 24 hours, after 1 hour of focal ischemia [47]. Maier et al. [9] confirmed that 1 and 2 hours of intra-ischemic mild hypothermia was effective to reduce apoptosis at 72 hours after the insult [9]. Similar results on infarct volume have been reported. For instance, Maier et al. [19] showed, 3 days after the insult, that 2 hours of hypothermia reduced the infarct volume, even when delayed up to 90 minutes after 2 hours MCAO [19]. In the same MCAO model, 4 hours of post-ischemic hypothermia (started 4 hours after ischemia onset), could no longer protect the rat brain [48,49]. However, a study by Ohta [50] showed that the hypothermic treatment could be delayed up to 4 hours after 2 hours MCAO if cooling was prolonged for 48 hours [50]. However, serious side effects are then to be expected, but were not investigated.

In our study, the number of apoptotic cells correlates well with infarct volume (r^2^ = 0.96) and with neurological deficit (r^2^ = 0.99). This observation is in accordance with the general idea that cell death in the penumbra, which is the predominant area that is salvaged by hypothermia, occurs through apoptosis [19-21,44]. It is also coherent with our hypothesis that the beneficial effects of short hypothermic treatment on infarct volume and neurological deficit are predominantly mediated by inhibition of apoptosis in the penumbra, at least as established at 24 hours after the insult.

After infusion of Et-1, CD-68 expression increased in the striatum and the cortex of the insult after 24 hours. These results are consistent with previous findings in the Et-1 model [37] and other MCAO models [51-53]. The hypothermic treatment significantly attenuated this increase by approximately 50%, when initiated 20 minutes after the infusion of Et-1. Similar results were obtained in studies investigating the effect of 2 hours of intra-ischemic mild hypothermic treatment after 2 hours MCAO. They showed a reduction of the infarct volume and CD-68 expression at 1, 3 and 7 days after the insult [15,16,22]. Similar results were also observed after MCAO of 8 minutes [54]. Our study further showed that delaying hypothermia for 1 or 2 hours could still reduce the amount of phagocytic cells in the core of the insult. Surprisingly, this observation did not correlate with decreased neurological or functional deficit. This could mean that a reduction in phagocytosis is not instrumental to the neuroprotective effects of hypothermia. However, it is conceivable that inhibition of microglial activation and reduced infiltration of monocytes within the first 24 hours attenuates the induction of cell death between 24 and 72 hours after the insult.

GFAP is considered the best marker to investigate reactive gliosis [55]. Stroke induces a massive increase in GFAP expression after a day, in several stroke models ranging from 30 minutes MCAO to the photothrombosis model [56,57]. Our study confirms these results in the Et-1 model, in the core as well as in the penumbra. Our major finding is that short hypothermic treatment reduced the expression of GFAP in the striatum and cortex. More specifically, Zoli et al. [57] described peak levels in astrogliosis 24 hours after the insult, especially in the penumbral area [57]. Our results are consistent with these findings and might explain why the effect of hypothermia on GFAP expression was less pronounced compared to that in the core of the insult. Although the strongest effects were found when hypothermia was initiated after 20 minutes, delaying hypothermia up to 2 hours after the administration of Et-1 still reduces GFAP expression. Further research is necessary to clarify whether delaying short hypothermic treatment for 2 hours (or more) provides neuroprotection at later time points after the insult. However, it is also possible that increased GFAP expression after cerebral ischemia is not entirely detrimental. Indeed, GFAP null mice showed more sensitivity to ischemia compared to wild type mice [58,59].

This study confirms that short hypothermic treatment can be protective when it is applied within a relatively short therapeutic window up to 1 h. It is known that, when the treatment is delayed, long cooling periods seem more protective [34,35]. In a balance of risk and benefit, a short duration of hypothermia may be the initial choice. The hypothermia treatment protocol should be tailored to each patient's situation, depending on the time after the insult and whether the patient is in the intra-ischemic or in the post-ischemic period. For instance, shorter cooling periods can be induced when patients arrive very quickly in the hospital after the onset of the insult, while longer cooling periods can be applied to patients that arrive later to the hospital. Also co-morbidities should be taken into account when conducting experimental or clinical studies [13]. Based on this assumption and the results of the available data, a larger randomized, controlled clinical trial of hypothermia in acute ischemic stroke is warranted.

## Conclusion

Administration of Et-1 resulted in an ischemic stroke associated with neurological deficit and a large infarct volume, 24 hours after the insult. Furthermore, apoptosis and gliosis were stimulated. Two hours of a short mild hypothermic treatment, initiated after 20 or 60 minutes improved neurological deficit and infarct volume, and coincided with a reduction in apoptotic cells. Glial activation, on the contrary, remained inhibited even when hypothermia was only induced after 2 hours. These data indicate that in the Et-1 rat model, a short mild hypothermic treatment delayed for 1 hour is still neuroprotective and correlates with apoptosis. At the same time, hypothermia also establishes a lasting inhibitory effect on the activation of astrogliosis.

## Competing interests

The authors declare no conflict of interest.

## Authors’ contributions

TZ, A-GC, SS, YM, RK and SH participated in the design of the study. TZ, A-GC carried out the data acquisition. In particular, A-GC participated. SS, RK, SH and YM provided writing assistance. In particular, SS participated in the statistical analysis. All authors read and approved the final manuscript.

## References

[B1] Candelario-Jalil E Injury and repair mechanisms in ischemic stroke: considerations for the development of novel neurotherapeutics Curr Opin Investig Drugs 2009 10 7 644 654 19579170

[B2] Lyden PD Krieger D Yenari M Dietrich WD Therapeutic hypothermia for acute stroke Int J Stroke 2006 1 1 9 19 10.1111/j.1747-4949.2005.00011.x 18706063

[B3] Zhao H Steinberg GK Sapolsky RM General versus specific actions of mild-moderate hypothermia in attenuating cerebral ischemic damage J Cereb Blood Flow Metab 2007 27 12 1879 1894 10.1038/sj.jcbfm.9600540 17684517

[B4] Hachimi-Idrissi S Corne L Ebinger G Michotte Y Huyghens L Mild hypothermia induced by a helmet device: a clinical feasibility study Resuscitation 2001 51 3 275 281 10.1016/S0300-9572(01)00412-9 11738778

[B5] Liu L Yenari MA Therapeutic hypothermia: neuroprotective mechanisms Front Biosci 2007 12 816 825 10.2741/2104 17127332

[B6] De Georgia MA Krieger DW Abou-Chebl A Devlin TG Jauss M Davis SM Koroshetz WJ Rordorf G Warach S Cooling for Acute Ischemic Brain Damage (COOL AID): a feasibility trial of endovascular cooling Neurology 2004 63 2 312 317 10.1212/01.WNL.0000129840.66938.75 15277626

[B7] Hemmen TM Raman R Guluma KZ Meyer BC Gomes JA Cruz-Flores S Wijman CA Rapp KS Grotta JC Lyden PD Intravenous thrombolysis plus hypothermia for acute treatment of ischemic stroke (ICTuS-L): final results Stroke 2010 41 10 2265 2270 10.1161/STROKEAHA.110.592295 20724711 PMC2947593

[B8] Colbourne F Corbett D Zhao Z Yang J Buchan AM Prolonged but delayed postischemic hypothermia: a long-term outcome study in the rat middle cerebral artery occlusion model J Cereb Blood Flow Metab 2000 20 12 1702 1708 11129786 10.1097/00004647-200012000-00009

[B9] Maier CM Ahern K Cheng ML Lee JE Yenari MA Steinberg GK Optimal depth and duration of mild hypothermia in a focal model of transient cerebral ischemia: effects on neurologic outcome, infarct size, apoptosis, and inflammation Stroke 1998 29 10 2171 2180 10.1161/01.STR.29.10.2171 9756600

[B10] Hammer MD Krieger DW Acute ischemic stroke: is there a role for hypothermia? Cleve Clin J Med 2002 69 10 770 773–774, 776–777 passim 10.3949/ccjm.69.10.770 12371800

[B11] Marion D Bullock MR Current and future role of therapeutic hypothermia J Neurotrauma 2009 26 3 455 467 10.1089/neu.2008.0582 19292697

[B12] Schwab S Georgiadis D Berrouschot J Schellinger PD Graffagnino C Mayer SA Feasibility and safety of moderate hypothermia after massive hemispheric infarction Stroke 2001 32 9 2033 2035 10.1161/hs0901.095394 11546893

[B13] Zgavc T Ceulemans AG Sarre S Michotte Y Hachimi-Idrissi S Experimental and clinical use of therapeutic hypothermia for ischemic stroke: opportunities and limitations Stroke Res Treat 2011 2011 689290 21789271 10.4061/2011/689290 PMC3140058

[B14] Bell TE Kongable GL Steinberg GK Mild hypothermia: an alternative to deep hypothermia for achieving neuroprotection J Cardiovasc Nurs 1998 13 1 34 44 9785204 10.1097/00005082-199810000-00005

[B15] Deng H Han HS Cheng D Sun GH Yenari MA Mild hypothermia inhibits inflammation after experimental stroke and brain inflammation Stroke 2003 34 10 2495 2501 10.1161/01.STR.0000091269.67384.E7 12970518

[B16] Han HS Qiao Y Karabiyikoglu M Giffard RG Yenari MA Influence of mild hypothermia on inducible nitric oxide synthase expression and reactive nitrogen production in experimental stroke and inflammation J Neurosci 2002 22 10 3921 3928 12019311 10.1523/JNEUROSCI.22-10-03921.2002 PMC6757647

[B17] Kelly S Cheng D Steinberg GK Yenari MA Mild hypothermia decreases GSK3beta expression following global cerebral ischemia Neurocrit Care 2005 2 2 212 217 10.1385/NCC:2:2:212 16159068

[B18] Maier CM Sun GH Cheng D Yenari MA Chan PH Steinberg GK Effects of mild hypothermia on superoxide anion production, superoxide dismutase expression, and activity following transient focal cerebral ischemia Neurobiol Dis 2002 11 1 28 42 10.1006/nbdi.2002.0513 12460544

[B19] Maier CM Sun GH Kunis D Yenari MA Steinberg GK Delayed induction and long-term effects of mild hypothermia in a focal model of transient cerebral ischemia: neurological outcome and infarct size J Neurosurg 2001 94 1 90 96 10.3171/jns.2001.94.1.0090 11147904

[B20] Van Hemelrijck A Hachimi-Idrissi S Sarre S Ebinger G Michotte Y Post-ischaemic mild hypothermia inhibits apoptosis in the penumbral region by reducing neuronal nitric oxide synthase activity and thereby preventing endothelin-1-induced hydroxyl radical formation Eur J Neurosci 2005 22 6 1327 1337 10.1111/j.1460-9568.2005.04331.x 16190888

[B21] Van Hemelrijck A Vermijlen D Hachimi-Idrissi S Sarre S Ebinger G Michotte Y Effect of resuscitative mild hypothermia on glutamate and dopamine release, apoptosis and ischaemic brain damage in the endothelin-1 rat model for focal cerebral ischaemia J Neurochem 2003 87 1 66 75 10.1046/j.1471-4159.2003.01977.x 12969253

[B22] Wang GJ Deng HY Maier CM Sun GH Yenari MA Mild hypothermia reduces ICAM-1 expression, neutrophil infiltration and microglia/monocyte accumulation following experimental stroke Neuroscience 2002 114 4 1081 1090 10.1016/S0306-4522(02)00350-0 12379261

[B23] Yenari MA Zhao H Giffard RG Sobel RA Sapolsky RM Steinberg GK Gene therapy and hypothermia for stroke treatment Ann N Y Acad Sci 2003 993 54 68 discussion 79–81 10.1111/j.1749-6632.2003.tb07511.x 12853295

[B24] Zhang Z Sobel RA Cheng D Steinberg GK Yenari MA Mild hypothermia increases Bcl-2 protein expression following global cerebral ischemia Brain Res Mol Brain Res 2001 95 1–2 75 85 11687278 10.1016/s0169-328x(01)00247-9

[B25] Zhao H Wang JQ Shimohata T Sun G Yenari MA Sapolsky RM Steinberg GK Conditions of protection by hypothermia and effects on apoptotic pathways in a rat model of permanent middle cerebral artery occlusion J Neurosurg 2007 107 3 636 641 10.3171/JNS-07/09/0636 17886565

[B26] Zhao H Yenari MA Sapolsky RM Steinberg GK Mild postischemic hypothermia prolongs the time window for gene therapy by inhibiting cytochrome C release Stroke 2004 35 2 572 577 10.1161/01.STR.0000110787.42083.58 14726551

[B27] Dietrich WD Atkins CM Bramlett HM Protection in animal models of brain and spinal cord injury with mild to moderate hypothermia J Neurotrauma 2009 26 3 301 312 10.1089/neu.2008.0806 19245308 PMC2848835

[B28] Kollmar R Schwab S Hypothermia in focal ischemia: implications of experiments and experience J Neurotrauma 2009 26 3 377 386 10.1089/neu.2008.0564 19231916

[B29] Tang XN Liu L Yenari MA Combination therapy with hypothermia for treatment of cerebral ischemia J Neurotrauma 2009 26 3 325 331 10.1089/neu.2008.0594 19216635 PMC2752358

[B30] Callaway JK Knight MJ Watkins DJ Beart PM Jarrott B Delayed treatment with AM-36, a novel neuroprotective agent, reduces neuronal damage after endothelin-1-induced middle cerebral artery occlusion in conscious rats Stroke 1999 30 12 2704 2712 discussion 2712 10.1161/01.STR.30.12.2704 10583001

[B31] Macrae IM Robinson MJ Graham DI Reid JL McCulloch J Endothelin-1-induced reductions in cerebral blood flow: dose dependency, time course, and neuropathological consequences J Cereb Blood Flow Metab 1993 13 2 276 284 10.1038/jcbfm.1993.34 8436619

[B32] Sicard KM Fisher M Animal models of focal brain ischemia Exp Transl Stroke Med 2009 1 7 10.1186/2040-7378-1-7 20150985 PMC2820445

[B33] Windle V Szymanska A Granter-Button S White C Buist R Peeling J Corbett D An analysis of four different methods of producing focal cerebral ischemia with endothelin-1 in the rat Exp Neurol 2006 201 2 324 334 10.1016/j.expneurol.2006.04.012 16740259

[B34] Clark DL Penner M Orellana-Jordan IM Colbourne F Comparison of 12, 24 and 48 h of systemic hypothermia on outcome after permanent focal ischemia in rat Exp Neurol 2008 212 2 386 392 10.1016/j.expneurol.2008.04.016 18538766

[B35] Yanamoto H Nagata I Nakahara I Tohnai N Zhang Z Kikuchi H Combination of intraischemic and postischemic hypothermia provides potent and persistent neuroprotection against temporary focal ischemia in rats Stroke 1999 30 12 2720 2726 discussion 2726 10.1161/01.STR.30.12.2720 10583003

[B36] Schaller B Graf R Hypothermia and stroke: the pathophysiological background Pathophysiology 2003 10 1 7 35 10.1016/j.pathophys.2003.09.001 14643901

[B37] Ceulemans AG Zgavc T Kooijman R Hachimi-Idrissi S Sarre S Michotte Y Mild hypothermia causes differential, time-dependent changes in cytokine expression and gliosis following endothelin-1-induced transient focal cerebral ischemia J Neuroinflammation 2011 8 60 10.1186/1742-2094-8-60 21627837 PMC3127770

[B38] Paxinos G Watson G The Rat Brain in Stereotaxic Coordinates 1986 New York: Academic

[B39] Bogaert L Scheller D Moonen J Sarre S Smolders I Ebinger G Michotte Y Neurochemical changes and laser Doppler flowmetry in the endothelin-1 rat model for focal cerebral ischemia Brain Res 2000 887 2 266 275 10.1016/S0006-8993(00)02959-0 11134615

[B40] Garcia JH Wagner S Liu KF Hu XJ Neurological deficit and extent of neuronal necrosis attributable to middle cerebral artery occlusion in rats. Statistical validation Stroke 1995 26 4 627 634 discussion 635 10.1161/01.STR.26.4.627 7709410

[B41] Weston RM Jones NM Jarrott B Callaway JK Inflammatory cell infiltration after endothelin-1-induced cerebral ischemia: histochemical and myeloperoxidase correlation with temporal changes in brain injury J Cereb Blood Flow Metab 2007 27 1 100 114 10.1038/sj.jcbfm.9600324 16736051

[B42] Durukan A Tatlisumak T Acute ischemic stroke: overview of major experimental rodent models, pathophysiology, and therapy of focal cerebral ischemia Pharmacol Biochem Behav 2007 87 1 179 197 10.1016/j.pbb.2007.04.015 17521716

[B43] Parker JD Thiessen JJ Reilly R Tong JH Stewart DJ Pandey AS Human endothelin-1 clearance kinetics revealed by a radiotracer technique J Pharmacol Exp Ther 1999 289 1 261 265 10087013

[B44] Broughton BR Reutens DC Sobey CG Apoptotic mechanisms after cerebral ischemia Stroke 2009 40 5 e331 e339 10.1161/STROKEAHA.108.531632 19182083

[B45] Chaitanya GV Babu PP Activation of calpain, cathepsin-b and caspase-3 during transient focal cerebral ischemia in rat model Neurochem Res 2008 33 11 2178 2186 10.1007/s11064-007-9567-7 18338260

[B46] Lee SH Kim M Kim YJ Kim YA Chi JG Roh JK Yoon BW Ischemic intensity influences the distribution of delayed infarction and apoptotic cell death following transient focal cerebral ischemia in rats Brain Res 2002 956 1 14 23 10.1016/S0006-8993(02)03197-9 12426041

[B47] Phanithi PB Yoshida Y Santana A Su M Kawamura S Yasui N Mild hypothermia mitigates post-ischemic neuronal death following focal cerebral ischemia in rat brain: immunohistochemical study of Fas, caspase-3 and TUNEL Neuropathology 2000 20 4 273 282 10.1046/j.1440-1789.2000.00346.x 11211051

[B48] Kawai N Okauchi M Morisaki K Nagao S Effects of delayed intraischemic and postischemic hypothermia on a focal model of transient cerebral ischemia in rats Stroke 2000 31 8 1982 1989 discussion 1989 10.1161/01.STR.31.8.1982 10926967

[B49] Kawamura N Schmeichel AM Wang Y Schmelzer JD Low PA Multiple effects of hypothermia on inflammatory response following ischemia-reperfusion injury in experimental ischemic neuropathy Exp Neurol 2006 202 2 487 496 10.1016/j.expneurol.2006.07.012 16934252

[B50] Ohta H Terao Y Shintani Y Kiyota Y Therapeutic time window of post-ischemic mild hypothermia and the gene expression associated with the neuroprotection in rat focal cerebral ischemia Neurosci Res 2007 57 3 424 433 10.1016/j.neures.2006.12.002 17212971

[B51] Kim HJ Rowe M Ren M Hong JS Chen PS Chuang DM Histone deacetylase inhibitors exhibit anti-inflammatory and neuroprotective effects in a rat permanent ischemic model of stroke: multiple mechanisms of action J Pharmacol Exp Ther 2007 321 3 892 901 10.1124/jpet.107.120188 17371805

[B52] Yenari MA Han HS Influence of hypothermia on post-ischemic inflammation: role of nuclear factor kappa B (NFkappaB) Neurochem Int 2006 49 2 164 169 10.1016/j.neuint.2006.03.016 16750872

[B53] Zhang Z Chopp M Powers C Temporal profile of microglial response following transient (2 h) middle cerebral artery occlusion Brain Res 1997 744 2 189 198 10.1016/S0006-8993(96)01085-2 9027378

[B54] Webster CM Kelly S Koike MA Chock VY Giffard RG Yenari MA Inflammation and NFkappaB activation is decreased by hypothermia following global cerebral ischemia Neurobiol Dis 2009 33 2 301 312 10.1016/j.nbd.2008.11.001 19063968 PMC2737398

[B55] Pekny M Nilsson M Astrocyte activation and reactive gliosis Glia 2005 50 4 427 434 10.1002/glia.20207 15846805

[B56] Nowicka D Rogozinska K Aleksy M Witte OW Skangiel-Kramska J Spatiotemporal dynamics of astroglial and microglial responses after photothrombotic stroke in the rat brain Acta Neurobiol Exp (Wars) 2008 68 2 155 168 18511952 10.55782/ane-2008-1685

[B57] Zoli M Grimaldi R Ferrari R Zini I Agnati LF Short- and long-term changes in striatal neurons and astroglia after transient forebrain ischemia in rats Stroke 1997 28 5 1049 1058 discussion 1059 10.1161/01.STR.28.5.1049 9158649

[B58] Cordeau P Jr Lalancette-Hebert M Weng YC Kriz J Live imaging of neuroinflammation reveals sex and estrogen effects on astrocyte response to ischemic injury Stroke 2008 39 3 935 942 10.1161/STROKEAHA.107.501460 18258827

[B59] Nawashiro H Brenner M Fukui S Shima K Hallenbeck JM High susceptibility to cerebral ischemia in GFAP-null mice J Cereb Blood Flow Metab 2000 20 7 1040 1044 10908037 10.1097/00004647-200007000-00003

